# Lactate Dehydrogenase-B Is Silenced by Promoter Methylation in a High Frequency of Human Breast Cancers

**DOI:** 10.1371/journal.pone.0057697

**Published:** 2013-02-21

**Authors:** Nicola J. Brown, Sue E. Higham, Branko Perunovic, Mohammad Arafa, Sabapathy Balasubramanian, Ishtiaq Rehman

**Affiliations:** 1 Department of Oncology, The Medical School, University of Sheffield, Sheffield, United Kingdom; 2 Department of Histopathology, Royal Hallamshire Hospital, Glossop Road, Sheffield, United Kingdom; 3 Department of Human Metabolism, The Mellanby Centre for Bone Research, The Medical School, University of Sheffield, Sheffield, United Kingdom; University Medical Centre Utrecht, The Netherlands

## Abstract

**Objective:**

Under normoxia, non-malignant cells rely on oxidative phosphorylation for their ATP production, whereas cancer cells rely on Glycolysis; a phenomenon known as the Warburg effect. We aimed to elucidate the mechanisms contributing to the Warburg effect in human breast cancer.

**Experimental design:**

Lactate Dehydrogenase (LDH) isoenzymes were profiled using zymography. LDH-B subunit expression was assessed by reverse transcription PCR in cells, and by Immunohistochemistry in breast tissues. LDH-B promoter methylation was assessed by sequencing bisulfite modified DNA.

**Results:**

Absent or decreased expression of LDH isoenzymes 1-4, were seen in T-47D and MCF7 cells. Absence of LDH-B mRNA was seen in T-47D cells, and its expression was restored following treatment with the demethylating agent 5'Azacytadine. LDH-B promoter methylation was identified in T-47D and MCF7 cells, and in 25/ 25 cases of breast cancer tissues, but not in 5/ 5 cases of normal breast tissues. Absent immuno-expression of LDH-B protein (<10% cells stained), was seen in 23/ 26 (88%) breast cancer cases, and in 4/8 cases of adjacent ductal carcinoma *in situ* lesions. Exposure of breast cancer cells to hypoxia (1% O_2_), for 48 hours resulted in significant increases in lactate levels in both MCF7 (14.0 fold, p = 0.002), and T-47D cells (2.9 fold, p = 0.009), but not in MDA-MB-436 (-0.9 fold, p = 0.229), or MCF10AT (1.2 fold, p = 0.09) cells.

**Conclusions:**

Loss of LDH-B expression is an early and frequent event in human breast cancer occurring due to promoter methylation, and is likely to contribute to an enhanced glycolysis of cancer cells under hypoxia.

## Introduction

Breast cancer is the most common cancer diagnosis and the leading cause of cancer-related deaths in women worldwide [1]. Moreover, the disease incidence has increased over the past three decades [2]. Despite advances in early detection and treatment, around 30% of patients with early-stage breast cancer will develop recurrent disease, which often presents as metastasis and a poor prognosis [3].

Normal cells in the presence of oxygen (aerobic conditions), derive most of their energy from glycolysis coupled with oxidative phosphorylation which is a highly efficient process yielding 38 molecules of ATP for each molecule of glucose consumed [4]. Glucose enters into the cell and undergoes a series of steps to generate pyruvate, which then enters into the mitochondria where it is metabolized to CO_2_ and water with the formation of ATP. Furthermore, this process is facilitated by the presence of oxygen which activates oxidative phosphorylation and inhibits glycolysis (known as the Pasteur effect), [5], [6]. However, when oxygen is limited (anaerobic conditions), such as following prolonged exercise, pyruvate undergoes fermentation where it is converted to lactate by a reaction catalyzed by Lactate Dehydrogenase (LDH), [7], [8].

LDH is a tetrameric enzyme, containing 2 major subunits (A and B), encoded by 2 different genes, LDH-A and LDH-B [8], [9]. The LDH-A and LDH-B subunits associate as tetramers to form 5 different isoenzymes (LDH-1 to LDH-5), which are comprised of either subunits LDH-B4 (LDH-1); LDH-B3:A1 (LDH-2); LDH-B2:A2 (LDH-3), LDH-B1:A3 (LDH-4) and LDH-A4 (LDH-5) subunits. These LDH isoenzymes catalyze the interconversion of pyruvate and lactate, which is a key step in the glycolysis pathway as it also regenerates NAD^+^, necessary for continued glycolysis [10]. The LDH-5 isoenzyme which is composed solely of four LDH-A subunits (LHD-A4), catalyzes the reduction of pyruvate into lactate with unparalleled efficiency, particularly under anaerobic conditions [8], [11]. However, this function of LDH gradually decreases as the number of LDH-B subunits compared to the LDH-A subunits increase. Thus, the LDH-1 isoenzyme composed solely of four LDH-B subunits, efficiently favours the backward conversion of lactate into pyruvate [8],[10].

In contrast to normal cells, cancer cells derive a major source of their energy from glycolysis even in the presence of oxygen, despite the fact that glycolysis alone is an energetically inefficient process, yielding only 2 molecules of ATP per molecule of glucose [12], [13], [14]. This increased glycolysis even in the presence of oxygen was first described by Otto Warburg in the 1920's, and is now known as the Warburg effect or aerobic glycolysis [13], [14]. Activation of aerobic glycolysis is thought to be a mechanism used by cancer cells to liberate them from a dependency on oxygen for their ATP production, particularly under hypoxic tumour microenvironments [7]. Furthermore, this altered metabolism of cancer cells is thought to confer a selective advantage for their survival and proliferation [10].

Although the precise molecular mechanisms underlying the Warburg effect are incompletely understood, environmental (nutrients, hypoxia, acidity, etc), genetic and epigenetic factors are known to play a role [5], [14], [15], [16]. A role for genetic and /or epigenetic factors has also emerged from the finding that the glycolytic phenotype persists in cancer cells isolated from tumours growing *in vivo* [5]. Tumour cells have been shown to undergo aerobic glycolysis following oncogene activation (*myc*, *ras*, AKT), and tumour suppressor (p53, VHL) gene inactivation, which act to upregulate Hypoxia-Inducible Factor 1 alpha (HIF-1α) levels [5], [7]. Increased HIF-1α, in turn results leads to increased transcription of genes involved in glucose transport, glycolysis, and lactate formation through up-regulation of the LDH-A subunit. Interestingly, the LDH-A subunit has been found to be frequently up-regulated in clinical tumours and is often associated with disease progression and a poor prognosis [11], [17], [18]. In addition to the above mechanisms contributing to the Warburg effect, we and others have previously reported absence or decreased expression of the LDH-B subunit in prostate and gastric cancers, due to hyper-methylation of the LDH-B promoter [19], [20]. Recently, decreased expression of LDH-B has been associated with higher histological grade and perineural invasion in bladder cancer, suggesting that LDH-B may be related to tumour progression [21].

The aim of the current study was to elucidate the mechanism(s) contributing to the Warburg effect in human breast cancer. Panels of human breast derived cell lines representing various stages of breast cancer development and progression (non-malignant, pre-malignant, pre-invasive, invasive and metastatic disease), were profiled for expression of LDH isoenzymes using zymography. Absent or decreased expression of the LDH-B subunit was seen in T-47D and MCF7 cells, and the underlying mechanisms were investigated by sequencing the LDH-B promoter region in bisulfite modified DNA from the cell lines, and human breast cancer tissues. Furthermore, functional relevance of the absent or decreased expression of the LDH-B subunit was investigated by subjecting both LDH-B deficient and proficient cells to conditions of hypoxia, and measuring lactate levels as an indicator of their dependency on glycolysis.

## Materials and Methods

### Cell lines and culture

Human breast cancer cell lines MDA-MB-436, MDA-MB-231, MCF7, T-47D, and the prostate cancer cell line LNCaP were obtained from the American Type Culture Collection (ATCC), (http://www.atcc.org/). The MCF10A, MCF10AT and MCF10DCIS.com breast epithelial cell lines, representing normal (immortalized) breast epithelial cells, pre-malignant and pre-invasive ductal carcinoma *in situ* (DCIS) disease respectively [22], were a kind gift from the Barbara Ann Karmanos Cancer Institute, Detroit, Michigan, USA. The LNCaP-LN3 prostate cancer cell line used as a positive control was a kind gift from Dr. Curtis Pettaway (MD Anderson Cancer Centre, USA), [23]. The MCF7, MDA-MB-436 and MDA-MB-231 cells were maintained in RPMI-1640 media containing Glutamax, supplemented with 10% fetal calf serum and Penicillin/ Streptomycin (Gibco-BRL, Paisley, United Kingdom). The T-47D cells were cultured in DMEM containing Glutamax, supplemented with 10% fetal calf serum and Penicillin/ Streptomycin (Gibco-BRL). The MCF10A, MCF10AT and MCF10DCIS.com cells were cultured in DMEM/F12 Advanced media supplemented with Penicillin/ Streptomycin, 5% horse serum, 10 mM HEPES, 10 µg/ml insulin, 20 ng/ml EGF, 100 ng/ml cholera toxin and 0.5 µg/ml hydrocortisone. All cells were confirmed to be free from Mycoplasma using the EZ-PCR Mycoplasma test kit (Geneflow Ltd, UK).

### Short tandem repeat profiling of cells

In order to confirm the authenticity of representative breast cell lines, DNA from MCF7, T-47D and MCF10 was analyzed by Short Tandem Repeat (STR) profiling of 10 loci, at the University of Sheffield Core Genomics facility (http://genetics.group.shef.ac.uk/), using the StemElite^TM^ ID system (Promega, Product code: G9530), and the products run on a ABI 3730 DNA analyzer, according to the manufactures instructions. STR matches were searched using the LGC standards database (may 2012): http://www.lgcstandardsatcc.org/ATCCCulturesandProducts/CellBiology/STRProfileDatabase/tabid/986/Default.aspx. The STR profiles of the cells tested were as expected with 18/18 matching alleles, thus confirming their authenticity.

### Patient tissue material

Archived paraffin-embedded tissues obtained from patients having surgery for breast cancer at the Royal Hallamshire Hospital were used. Prior local ethics committee approval (South Yorkshire Research Ethics reference: 09/H1310/54), and informed patient consent was obtained in all cases. Genomic DNA was extracted from micro-dissected tissue sections as previously described [24], and used for sequence analysis. In addition, genomic DNA extracted from 5 cases of normal human breast tissue was purchased from Ambsbio (www.ambsbio.com), with patient ages ranging from 21-83 yrs. For sequencing of the LDH-B gene promoter region, 25 cases of adenocarcinoma were used which comprised of grade 1 (n = 5); grade 2 (n = 10), and grade 3 (n = 10) tumours. For immunohistochemistry, 26 cases of adenocarcinoma were used; 18 of which were also used for DNA sequence analysis.

### Zymography for lactate dehydrogenase isoenzymes

Zymography for LDH isoenzymes was performed using cellulose acetate membranes (Helena Biosciences, Titan III (94×76 mm)), with Tris-Glycine buffer according to the supplied manufacturer's instructions and as previously described [25]. Following electrophoresis, the gels were placed in 10 ml of staining solution containing 5 mM NAD, 50 mM lithium lactate, 0.1 mM Tris-HCl (pH 8.6), 0.2 mM Phenazinmethosulphate (PMS), 2.0 mM nitrobluetetrazolium (NBT), and 0.8 mM (3-(4,5-Dimethylthiazol-2-yl)-2,5-diphenyltetrazolium bromide (MTT), and the colour was allowed to develop (3- 6 min) in the dark at 37°C.

### Reverse transcription PCR

Total RNA was extracted using TRI Reagent (Sigma-Aldrich, Dorset, UK), according to the manufacturer's instructions. After precipitation, the RNA pellet was washed three times with 75% ethanol. RNA was quantified spectrophotometrically. RNA (2 µg) was reverse transcribed into cDNA using the SuperScript® III First-Strand Synthesis System (Invitrogen, Paisley, UK), with 250 ng of random primers, according to the manufacturer's instructions. LDH-B mRNA expression was assessed by reverse transcription PCR (RT-PCR), using primers and conditions as previously described [19].

### Sequencing of sodium bisulfite-modified DNA

DNA was extracted from breast cancer cell lines using the Gentra Puregene cell kit (Qiagen), according to the supplied manufacturer's instructions. Prior to sequencing, DNA was subject to sodium bisulfite-modification using the CpGenome^TM^ DNA modification kit (Invitrogen), according to the supplied manufacturer's instruction manual. For sequencing of the LDH-B promoter region, sodium bisulfite-modified DNA was amplified using previously published PCR primer sequences and conditions [19]. Only those cases showing an intense single PCR band following electrophoresis through a 2% agarose gel were excised and subject to sequence analysis. Sequencing of bisulfite modified DNA was performed at the University of Sheffield Core Genomics facility and the chromatograms visualized using the freely downloaded Chromas software (http://www.technelysium.com.au/chromas.html).

### Hypoxic cell culture

For the hypoxic cell culture experiments, T-47D, MCF7, MDA-MB-436, and MCF10AT cells were initially cultured under normoxic conditions as described above. Cells were then trypsinized and plated into T25cm^2^ flasks, and the flasks transferred into a normoxic (21% O_2_) incubator, or hypoxic (1% O_2_) chamber, as previously described [26]. The cell culture medium used for hypoxic growth had been pre-equilibrated to the same oxygen tension as in the hypoxic chamber for 6 hours. Lactate levels in the conditioned medium were measured following 48 hours of cell growth under hypoxic or normoxic conditions.

### Lactate measurements

Lactate concentrations in the conditioned medium were measured using a protocol available from the Sigma Aldrich website: http://www.sigmaaldrich.com/etc/medialib/docs/Sigma/Enzyme_Assay/llacticacidmw.Par.0001.File.tmp/llacticacidmw.pdf. Briefly, the assay depends on the reduction of NAD^+^ to NADH and a change in absorbance, which is proportional to the concentration of lactate in the medium. A standard curve was constructed using lactate concentrations ranging from (0–1000 µM lactate). In addition, lactate concentrations were assayed at the Department of Clinical Chemistry, Royal Hallamshire Hospital, Sheffield, using a Roche 501 analyzer, and the Lact2 lactate Gen.2 assay (Roche). Lactate levels were expressed as µM per million cells, by counting the final cell numbers using a coulter counter (Beckman Coulter model Z2), and after subtracting the values for the basal lactate levels present in the unconditioned media.

### Immunohistochemistry and scoring of staining

Immunohistochemistry for expression of the LDH-B protein in human breast tissue sections was performed as previously described, using 3,3'-Diaminobenzidine (DAB) as the chromogen [19]. The anti-LDH-B antibody used has previously been shown to be LDH-B specific and non-reactive against LDH-A [27]. Immunostained sections were assessed by 2 independent histopathologists (BP and MA), and the percentage of cells stained recorded. Relevant tissue areas scoring <10% of stained cells were recorded as showing absent LDH-B expression, while tissues showing ≥10% of stained cells were recorded as showing expression. Images were taken using a Nikon Eclipse 80i microscope attached to a Nikon digital camera (DS-2mv), and the images acquired using the Nikon Nis-Elements imaging software.

### 5'- Azacytidine treatment

T-47D cells were cultured in T75 cm^2^ flasks to ∼20% confluence. A final concentration of 1 µM freshly prepared 5'-azacytidine (Sigma-Aldrich), was added to the culture medium and the drug containing medium was changed every 2 days [19]. After 5 days of treatment, total RNA was isolated using TRI reagent (Sigma) as described above. The RNA was quantified and 200 ng was used for RT-PCR as described above.

### Statistical Analysis

All statistical analyses were performed using GraphPad Prism (version 5.02).

## Results

### LDH isoenzymes are absent or decreased in T-47D and MCF7 cells

**Figure 1:**
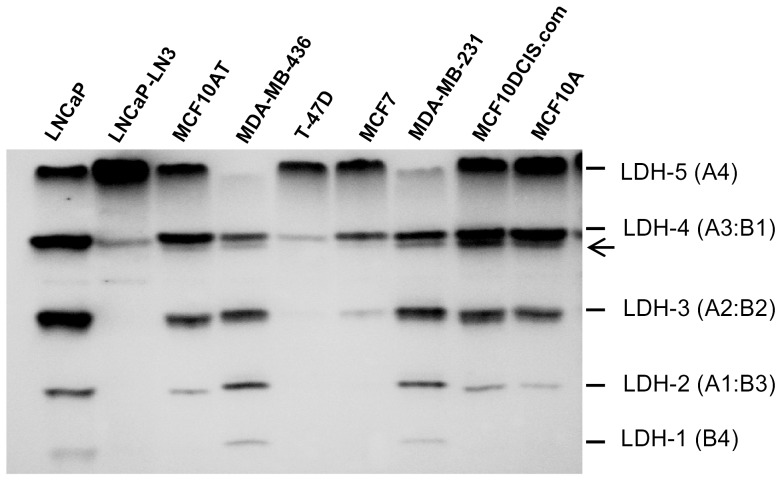
Zymography of human breast cell lines. Note the absent or decreased expression of LDH isoenzymes 1–4 in T-47D and MCF7 cells. In contrast, expression of LDH isoenzymes 1–4, can be seen in MDA-MB-436 and MDA-MB-231 cells. Expression of LDH isoenzymes 2-5, are predominant in the MCF10AT, MCF10DCIS.com and MCF10A cells. The expression of all 5 LDH isoenzymes can be seen in LNCaP cells, while absent or decreased expression of LDH isoenzymes 1–4, can be seen in the LNCaP-LN3 cells used as controls. In MCF10AT, MDA-MB-436, MDA-MB-231, MCF10DCIS.com and MCF10A cells, a weak band (*arrow head*), can be seen under the LDH-4 isoenzyme band, and may represent a posttranslationally modified form of LDH [28].

Following visualization of the stained zymography gel, absent or decreased expression of LDH isoenzymes 1–4 were seen in T-47D and MCF7 cells, with the isoenzyme pattern being similar to the LNCaP-LN3 prostate cells used as a control (Figure 1). In contrast, expression of LDH isoenzymes 1–4 were seen in both MDA-MB-436 and MDA-MB-231 cell lines, although these cells showed absent or reduced expression of the LDH-5 isoenzyme. Expression of LDH isoenzymes 2–5 were prominent in the MCF10AT, MCF10DCIS.com and MCF10A cells. Interestingly, in MCF10AT, MDA-MB-436, MDA-MB-231, MCF10DCIS.com and MCF10A cells, an additional band was visible just below the LDH-4 isoenzyme band (Figure 1, arrowhead). This may represent posttranslational modified forms of the LDH protein, since it has been reported that LDH may be subject to several posttranslational modifications such as phosphorylation, acetylation and methylation [28].

### LDH-B promoter is methylated in T-47D and MCF7 cells

**Figure 2:**
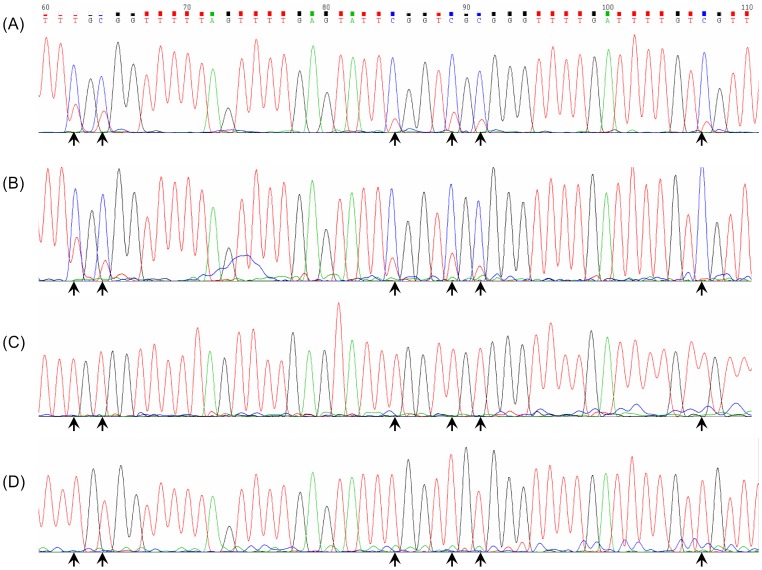
Sequencing chromatograms of sodium bisulfite modified DNA Methylation at CpG sites (*marked by arrowheads*), can be seen in (a) T-47D cells and in (b) a case of human breast cancer tissue by the presence of unmodified CpG residues (*arrowheads*). Identical sites can be seen to be modified and therefore unmethylated in (c) normal human breast tissue and (d) peripheral blood lymphocyte DNA from a patient with breast cancer.

In mammals, DNA methylation of cytosine residues located at 5' to guanine residues (CpG sites), represents a major epigenetic mechanism for regulating gene expression, including X-chromosome inactivation and genomic imprinting [29]. DNA hypermethylation has been shown to result in gene silencing with consequent loss of gene expression. To understand the mechanisms underlying the absent or decreased expression of the LDH-B subunit as seen in the T-47D and MCF7 cells, we sequenced the LDH-B promoter region in these and the other breast cell lines. Sequencing of bisulfite modified DNA showed methylation of the LDH-B promoter region in both T-47D and MCF7 cells (Figure 2a), while the LDH-B expressing cells; MDA-MB-436, MDA-MB-231, MCF10A, MCF10AT, were all seen to be unmethylated.

### LDH-B promoter methylation is frequent in breast cancer tissues

Sequencing of the LDH-B promoter region in DNA extracted from 25 cases of human breast cancer tissues of various grades (1–3), showed evidence of LDH-B promoter methylation in all 100% (25/25) cases (Figure 2b). Careful examination of all 14 of the CpG sites within the amplifed promoter region, determined that all of the sites showed evidence of methylation in tumour tissues and the methylated breast cancer cell lines. In contrast, LDH-B promoter methylation was absent in all 5 cases of normal breast tissue DNA (Figure 2c), in peripheral blood lymphocytes from matched (n = 10), or unmatched patients (n = 15) (Figure 2d), and in normal breast skin epithelium (n = 13), used as control tissues. In these cases, an absence of methylation was noted at all 14 of the CpG sites within the promoter region analysed.

### LDH-B protein expression is absent in breast cancer and pre-invasive lesions

**Figure 3:**
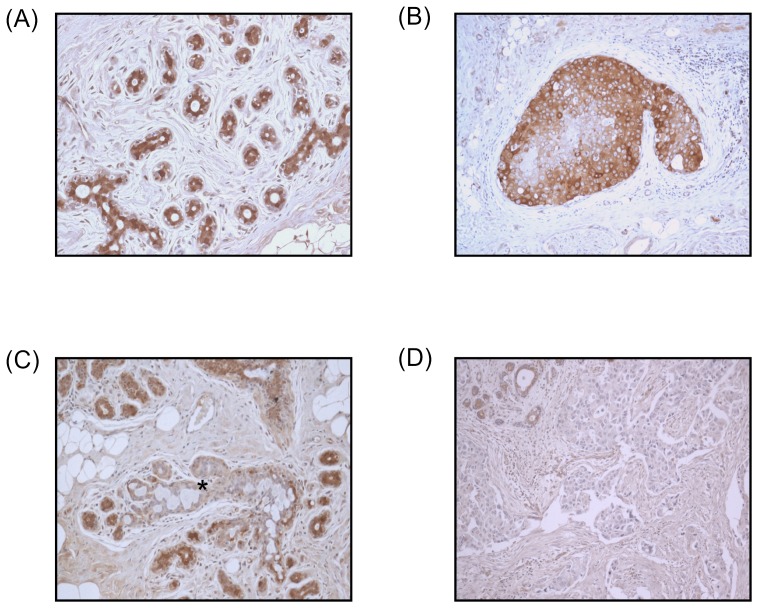
Immunoexpression of LDH-B in human breast tissues. (a) Strong cytoplasmic expression of LDH-B protein can be seen in the glands of non-malignant epithelium (*x20 magnification*), and (b) in a case of pre-invasive DCIS lesion (*x10*). (c) A DCIS lesion (*marked by asterix*) showing absent expression of LDH-B, while strong expression can be seen in the adjacent and surrounding non-malignant glands (*x10*). (d) A breast cancer case showing absent LDH-B expression, while expression can be seen in non-malignant glands at the top left of the image (*x10*).

Immunoexpression of the LDH-B protein was assessed in 26 cases of breast cancer tissues, which in some cases included adjacent pre-invasive DCIS lesions and non-malignant glands. Following microscopic examination, LDH-B protein expression was seen to be localized to within the cytoplasm of non-malignant glands, in 4/ 8 cases of DCIS lesions and in 3/ 26 cases (12%), of breast cancers (Figure 3a & 3b). However, absent expression of LDH-B protein expression was seen in 23/26 (88%) cases of breast cancer, and in 4/8 (50%) cases of adjacent DCIS lesions (Figure 3c & 3d).

### Re-expression of LDH-B mRNA by 5'-azacytidine treatment

**Figure 4:**
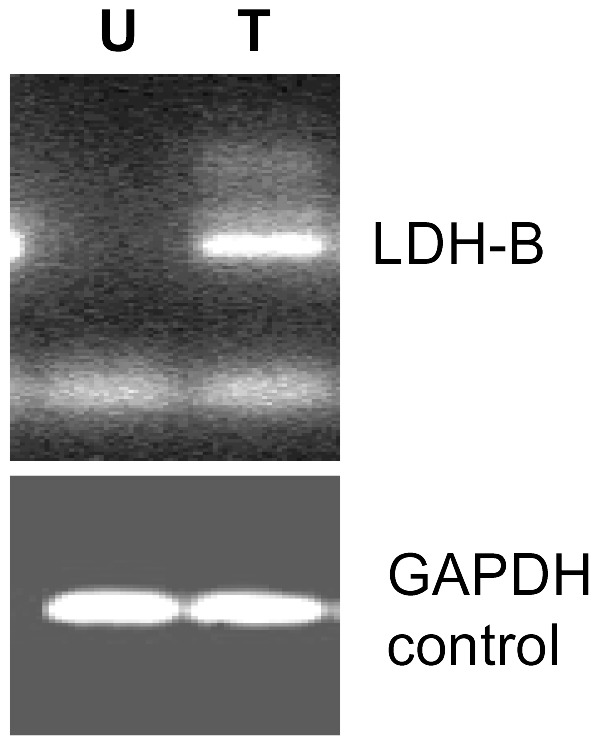
Reverse-transcription PCR for LDH-B mRNA. Untreated (U) T-47D cells showed an absence of LDH-B mRNA expression. Treatment (T) of cells with 1 µM 5'-Azacytidine for 5 days, was able to restore mRNA expression. GAPDH was used as a loading and amplification control.

We next tested the ability of the DNA methyltransferase inhibitor, 5'-Azacytidine [30], to restore expression of the LDH-B mRNA in T-47D cells, which were seen not to express LDH-B mRNA by RT-PCR (Figure 4). Treatment of T-47D cells with 5'-Azacytidine for 5 days showed re-expression of the LDH-B mRNA.

### Hypoxia leads to increased lactate levels in LDH-B deficient breast cancer cells

**Figure 5:**
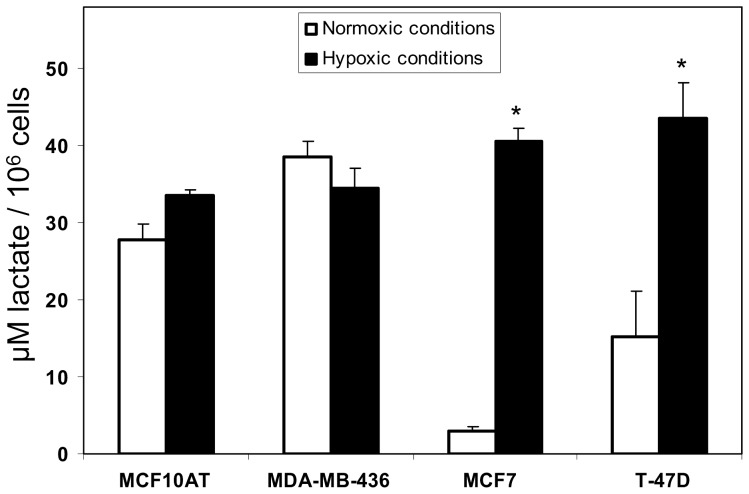
Lactate levels in conditioned medium from breast cell lines under normoxia and hypoxia. Following hypoxic growth, mean lactate levels in the conditioned medium from MCF10AT cells increased from 27.9 µM to 33.6 µM per 1×10^6^ cells (1.2 fold change), but this difference was not statistically significant (p = 0.09, Mann Whitney test). In MDA-MB-436 cells the mean lactate levels decreased slightly from 38.5 µM to 34.5 µM per 1×10^6^ cells (-0.9 fold change), but was not statistically significant (p = 0.229). However, following hypoxic growth of MCF7 cells, mean lactate levels increased significantly (p = 0.002), from 2.9 µM to 40.7 µM per 1×10^6^ cells (14.0 fold change). Similarly, in T-47D cells the mean lactate levels were found to increase significantly (p = 0.009), from 15.2 µM to 43.5 µM per 1×10^6^ cells (2.9 fold change), following hypoxic growth. * indicate statistically significant differences (p<0.05).

It has previously been reported that the LDH-A subunit is highly efficient at converting pyruvate to lactate particularly under hypoxic conditions [8], [16]. We therefore investigated the effects of hypoxia on both LDH-B promoter methylated (T-47D and MCF7), and unmethylated (MDA-MB-436, MCF10AT) cells, as the T-47D and MCF7 cells were seen to predominantly express the LDH-A4 (LHD-5) isoenzyme. All 4 cell lines were cultured under conditions of normoxia (21% O_2_), or hypoxia (1% O_2_) for 48 hours, and lactate levels were measured in the conditioned medium as described above. Following hypoxic growth, mean lactate levels in the conditioned medium from MCF10AT cells increased from 27.9 µM to 33.6 µM per 1×10^6^ cells (1.2 fold change), but this difference was not statistically significant (p = 0.09, Mann Whitney test), (Figure 5). In MDA-MB-436 cells the mean lactate levels decreased slightly from 38.5 µM to 34.5 µM per 1×10^6^ cells (-0.9 fold change), but was not statistically significant (p = 0.229). However, following hypoxic growth of MCF7 cells, mean lactate levels increased significantly (p = 0.002), from 2.9 µM to 40.7 µM per 1×10^6^ cells (14.0 fold change). Similarly, in T-47D cells the mean lactate levels were found to increase significantly (p = 0.009), from 15.2 µM to 43.5 µM per 1×10^6^ cells (2.9 fold change) following hypoxic growth (Figure 5).

**Figure 6:**
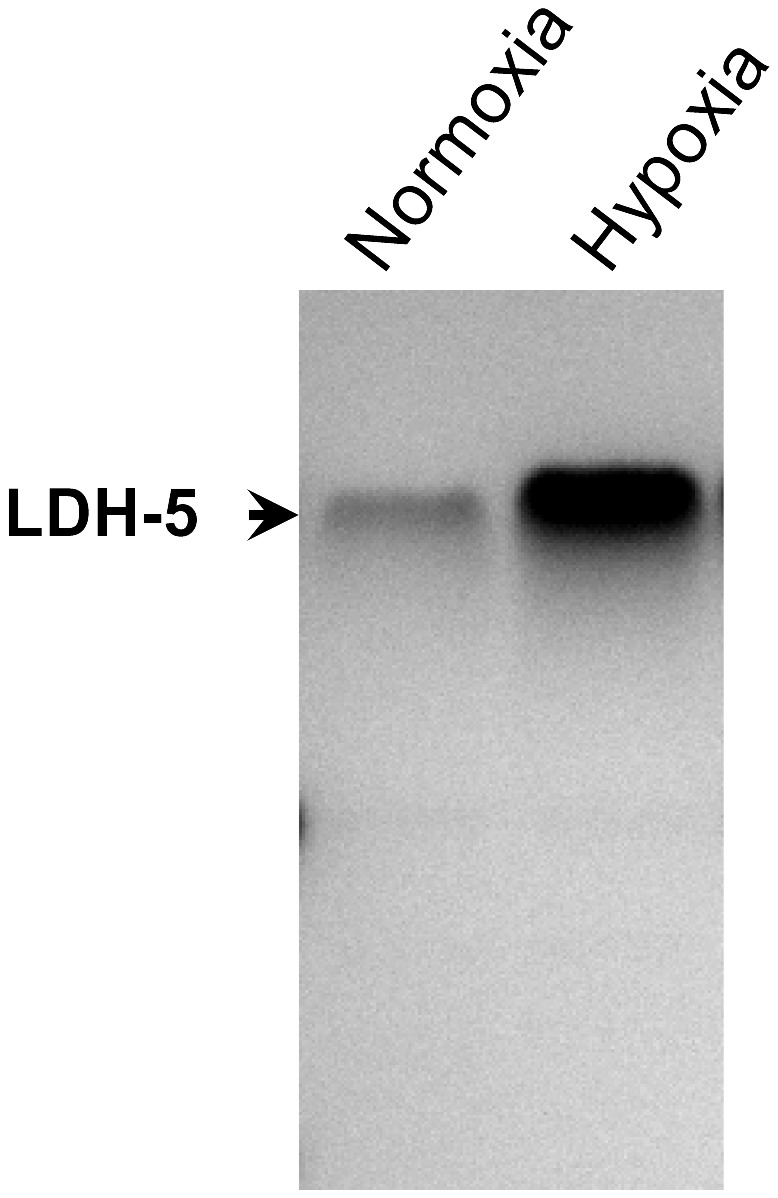
Zymography of T-47D cancer cells following hypoxic treatment. Note the relative increased activity of LDH-5 following hypoxic growth.

In order to determine the effects of hypoxia on LHD-5 isoenzyme activity, T-47D cells were cultured under both normoxic and hypoxic conditions and subjected to zymography analysis. As expected, the LDH-5 isoenzyme activity was seen to increase following hypoxic growth (Figure 6), since hypoxia has previously been shown to elevate expression of the LDH-A subunit [11], [17].

## Discussion

It has been known since the 1920's that cancer cells derive a major source of their energy by aerobic glycolysis; a phenomenon known as the Warburg effect, in contrast to normal cells which rely mainly on oxidative phosphorylation [6], [12], [14]. Although the precise mechanism(s) for the altered metabolism in cancers is unknown, previous studies have demonstrated diverse mechanisms, including oncogene activation, tumour suppressor gene inactivation, hypoxia, up-regulation of the LDH-A subunit, and absent or decreased expression of the LDH-B subunit [5], [14], [15], [16], [19]. Our current study has shown for the first time in human breast cancer that loss of expression of the LDH-B subunit may occur at an early (DCIS) stage, and occurs in a high frequency of breast cancers, with the underlying mechanism likely to involve methylation of the LDH-B promoter. Furthermore, since the LDH-B deficient cells showed significantly increased lactate levels when exposed to hypoxic conditions, the loss or reduction in LDH-B expression may provide these cancer cells with a growth and survival advantage. In addition, the finding of absent or reduced expression of the LDH-B subunit in 2 widely used human breast cancer cell lines (T-47D and MCF7), could have consequences for data interpretation during the use of these cell lines in metabolic or other studies.

By investigating a range of breast cell lines for their LDH expression profile, we have shown that the T-47D and MCF7 cells predominantly express the LDH-5 (LDH-A4) isoenzyme, with relatively low levels of the LDH-4 (LDH-A3:B1) isoenzyme. Although our results are in stark contrast to the data reported by Hussien and Brooks (2011), who showed that MCF7 cells expressed mainly the LDH-B subunit [31], our data are in partial agreement with those reported by Burke *et al*., (1978), who showed that MCF7 cells expressed only the LDH-5 isoenzyme [32]. The difference between our data and those reported by Burke *et al*., (1978), may be related to technical factors such as differences in the sensitivity of the staining procedure, amounts of protein loaded or the possibility that the LDH-B gene promoter may have become unmethylated in a small proportion of MCF7 cells to allow re-expression of the LDH-B protein, which could explain the relatively low level expression of the LDH-4 isoenzyme seen in the MCF7 cells (Figure 1). The possibility that the MCF7 cells were contaminated with another cell line can be firmly ruled out from our STR profiling data, which authenticated these cells.

Due to the absent or decreased expression of the LDH-B subunit, T-47D and MCF7 cells were seen to express predominantly the LDH-5 (LDH-A4), and LDH-4 (LDH-A3:B1) isoenzymes, which are enriched for the LDH-A subunit. This data is in agreement with a previous study showing that LDH-A is the predominant isoform expressed in breast tissue [33]. Other studies have demonstrated a critical role for LDH-A in promoting the growth of breast and other cancer types. For instance, silencing the expression of LDH-A has been shown to decrease cellular proliferation and tumourigenic ability of breast cancer cells [34], and furthermore LDH-A has been shown to be involved in tumour progression [35].

Previous studies have reported that the glycolytic rate in cultured cells appears to correlate with tumour aggressiveness [5]. Thus, it has been reported that the non-invasive MCF7 cells have a much lower aerobic glucose consumption rate compared with the highly invasive MDA-MB-231 breast cancer cell line [5]. Although this finding is in line with our data where we found that during conditions of normoxia, lactate levels were higher in the highly invasive MDA-MB-436 cells compared with MCF7 cells, we did not expect this finding based on the LDH isoenzyme profiles observed in these cells. Since the MCF7 cells are deficient in LDH-B, even under normoxic conditions they were expected to show higher levels of lactate production due to their expression of LDH-A, which is the subunit that has the highest efficiency for the conversion of pyruvate to lactate [8], [10]. These findings suggest that there are factors other than the loss of LDH-B expression, which may underlie the Warburg effect in human breast cancer.

Tumour cells are periodically exposed to hypoxic and normoxic environments [5]. Following hypoxic growth, we measured significant changes in lactate levels in the LDH-B deficient MCF7 and T-47D cells, whereas the LDH-B proficient MCF10AT and MDA-MB-436 cells showed insignificant changes, compared to normoxic growth conditions (Figure 5). Thus, our data show that cells with absent or decreased expression of LDH-B (but express LDH-A), may be better adapted to hypoxic growth by up-regulating fermentative glycolysis. Even under normoxic conditions, we were expecting breast cancer cells with absent or decreased expression of LDH-B to show relatively higher levels of lactate production [8], [10]. However, we observed the opposite result, in that cells with absent or decreased LDH-B expression had relatively low levels of lactate production under normoxic conditions. Thus, our data suggest that under normoxic growth conditions, breast cancer cells which express both LDH-A and LDH-B subunits rely on glycolysis for their energy production, as evidenced by the relatively higher rates of lactate production, and this is maintained even under hypoxic conditions. In contrast, cells with absent or decreased expression of LDH-B (but express LDH-A), under normoxic conditions rely mainly on non-glycolytic pathways for their energy production, but under hypoxic conditions may switch to fermentative glycolysis for their energy production. This data is in keeping with other published data suggesting that certain tumours may rely more on non-glucose carbon sources, even in glucose replete conditions [36].

Previous studies have shown that lactate plays a number of key roles in cancer development, progression and metastasis [10], [37], [38]. For instance, in colorectal cancer a study has shown that lactate can be extruded from cancer cells into the extracellular matrix and subsequently taken up by tumour-associated stromal fibroblasts to be used as a fuel [39]. Additionally, lactate may constitute an alternative metabolic fuel for cancer cells, as oxidative tumour cells can use lactate in preference to glucose, thereby sparing glucose to fuel hypoxic tumour cell growth [37]. In tumours with increased glycolysis and consequently increased lactate production, the extracellular pH has been shown to be acidic [40], [41]. Furthermore, a low pH has been shown to stimulate *in vitro* invasion and *in vivo* metastasis of cancer cells [42]. Clinical based studies in patients with cervical cancer or head and neck squamous cell cancer (HNSCC), have shown lactate levels to be inversely correlated with patient survival [38]. Thus, our finding of increased lactate levels in breast cancer cells with absent or reduced expression of the LDH-B subunit when exposed to hypoxic conditions, suggests that the increased lactate levels may contribute to fuel aggressive tumour behavior.

The glycolytic phenotype has been shown to coincide with the transition from pre-malignant lesions to invasive cancer [5]. This finding is in agreement with our data from breast cancer, since by immunohistochemistry we found LDH-B protein to be expressed in 4/ 8 pre-invasive DCIS lesions, but an absence of expression in the adjacent cancer. However, LDH-B protein expression was found to be absent in the remainder 4/ 8 cases of DCIS lesions, which suggests that the loss of LDH-B protein expression may occur earlier than previously reported i.e. during the transition from non-malignant epithelium to pre-invasive DCIS lesions. In support of this, Ong and colleagues reported an increase in glucose consumption in pre-malignant intestinal polyps and cancers compared with non-malignant mucosa [43].

Despite the gaps in our knowledge concerning the glycolytic phenotype, our data showing absent or decreased expression of the LDH-B subunit, may open up a new therapeutic strategy for the treatment of breast and other cancer types. Targeting the remaining LDH-A subunit with therapeutic agents, specifically in those tumours with an absent or decreased expression of LDH-B is expected to lead to a greater cell death, as this is expected to inhibit all cellular LDH activity, and lead to a depletion of the NAD^+^ stock necessary for self-sufficient glycolysis to occur. In support of this idea, a recent study has shown that specifically targeting the LDH-A subunit using a small molecule inhibitor (FX11), or by siRNA knock-down, increased cell death and inhibited the progression of tumours *in vivo* [35]. Furthermore, MCF7 cells (with decreased expression of LDH-B), were highly sensitive to growth inhibition by FX11 [35]. Interestingly studies on naturally occurring LDH-A null patients have shown that such patients do not usually develop symptoms during normal physiological conditions, although excessive fatigue and myoglobulinuria have been noted after intense anaerobic exercise [44]. Taken together with published data, our study provides promising evidence for the possibility that the LDH-A subunit may be a safe 'drugable' target for the development of novel anti-cancer agents [45]. Future efforts should therefore be focused on developing non-toxic and highly specific small molecule inhibitors of the LDH-A subunit, in order to block glycolysis, particularly in those tumours which have absent or decreased expression of the LDH-B subunit, thus, 'cutting-off' a major pathway used by breast and other cancer cells for their energy production.
